# Access to inclusive sanitation and participation in sanitation programs for people with disabilities in Indonesia

**DOI:** 10.1038/s41598-023-30586-z

**Published:** 2023-03-15

**Authors:** D. Daniel, Anindrya Nastiti, Hana Yesica Surbakti, Ni Made Utami Dwipayanti

**Affiliations:** 1 Department of Health Behavior, Environment, and Social Medicine, Faculty of Medicine, Public Health and Nursing, Universitas Gadjah Mada, Yogyakarta, Indonesia; 2 Faculty of Civil and Environmental Engineering, Institut Teknologi Bandung, Bandung, Indonesia; 3 Center for Environmental Studies, Institut Teknologi Bandung, Bandung, Indonesia; 4 Department of Public Health, The Graduate School, Universitas Gadjah Mada, Yogyakarta, Indonesia; 5 Department of Public Health and Preventive Medicine, Faculty of Medicine, Udayana University, Denpasar, Indonesia

**Keywords:** Environmental social sciences, Psychology and behaviour

## Abstract

Access to inclusive sanitation for people with disabilities (PWDs) remains a global challenge, including in Indonesia, where 10–15% of its population is disabled. Inclusive sanitation facilities can be achieved when PWDs are involved in the sanitation-related decision-making process, e.g., designing toilet that meets their needs. This study aims to investigate the situation of the sanitation facility in houses of PWDs and understand knowledge, attitude, and practices related to PWD participation in sanitation programs using a case study in two provinces in Indonesia: Nusa Tenggara Barat and Nusa Tenggara Timur. Quantitative data were taken from 129 PWD households, and qualitative data were from in-depth interviews with relevant stakeholders. The latter was presented in a causal loop diagram. Less than 5% had inclusive sanitation at home, mainly due to no supportive tools and bins. The knowledge levels related to sanitation and PWDs participation in sanitation programs were relatively low. Economic condition was one of the barriers for PWDs to participate in the sanitation program. Statistical analysis found that a higher household head education level was associated with a more positive attitude and higher PWD participation in sanitation programs. Although Indonesian law supports PWD participation, the implementation remains a challenge. This study also underlines the need for capacity building in facilitating PWDs’ involvement in sanitation programs and community meetings. Finally, barriers to PWD participation can come from different levels and actors, e.g., the family, the community, the district level, and the PWD itself, indicating the need to involve actors at all levels to enhance PWD participation in the sanitation program that leads to inclusive sanitation facilities for all groups.

## Introduction

Sanitation, together with hygiene and safe water (WASH), is essential for public health protection and social and economic development. Poor sanitation is a major risk factor for diarrhoea, neglected tropical diseases, and malnutrition^1^. Target 6.2 of the Sustainable Development Goals calls for universal access to adequate and equitable sanitation. Although the population practising open defecation decreased by one-third from 2015 to 2020, 3.6 billion people, or nearly half of the world’s population, still lacked safely managed sanitation services^2^. These global aggregate figures may also mask population vulnerabilities. The WHO/UNICEF Joint Monitoring Programme (JMP) 2021 reported that access to sanitation varies by wealth quintiles, with open defecation being more prevalent among the poorest segment of society. Despite being recognised as a human right and a prerequisite of sustainable development, the sanitation sector still suffers from inequity issues.

Target 6.2 also calls to pay special attention to the needs of those in vulnerable situations, highlighting the phrase “to leave no one behind”^3^, including people with disability (PWD). The 2006 Convention on the Rights of Persons with Disabilities recognised that disability is caused by interactions between physical disabilities and behavioural and environmental barriers that prevent them from fully and effectively participating in society on an equal basis with others^4^. PWDs constitute 15% of the world’s population, or over 1 billion people^5^. These particularly vulnerable populations often face difficulties and discrimination while accessing health and health-related services, including WASH^6^.

There exists a growing body of evidence linking WASH access and PWDs. A cross-sectional survey in four countries suggests that access to WASH facilities is of inferior quality for PWDs^7^. In Nepal, PWDs had significantly more trouble accessing WASH than people without disabilities; PWDs have difficulty using WASH facilities independently, hygienically, and without discomfort or fear of abuse^8^. PWDs also suffer from inadequate WASH facilities in public settings, e.g., Indonesia’s tourism sector^9^. These burdens are higher for women and girls with disabilities^6^. Some structural obstacles hinder PWDs’ access to WASH facilities. Design and hardware issues are not the only obstacles for PWDs. Social barriers vary across cultures; however, studies from several nations indicate that PWDs are frequently stigmatised and discriminated against by others when using household and public facilities^10^.

The prevalence of PWDs in Indonesia ranges between 10 and 15%^11^, which is relatively similar to the global situation, i.e., 15%^12^. The proportion of PWDs in Indonesia is as follows: 15.9% were blindness; 10.5% were deafness; 7.1% were mute; 3.5% were muteness and deafness; 33.8% had a physical disability; 13.7% had mental retardation; 7% had a physical disability and mental retardation or other multiple disabilities; and 8.5% had a mental disability^13^. The 2008 Indonesian Central Statistics Agency survey indicates that PWDs are at risk of poverty^13^. Disability increases the likelihood of falling below the national poverty level^14^. Moreover, PWDs have continuously lower levels of educational achievement, health outcomes, and access to public services than people without disabilities^14^.

PWDs in Indonesia have witnessed a substantial shift in how disability is discussed and positioned in public discourse. The language used to describe PWDs has evolved at the most fundamental level. The word *”cacat*” in Indonesian, which means deformity, has been replaced with “*disabilitas”* or “*difabel,*” which implies disability or diffability^11,15^. Further, Act Number 8 of 2016 on persons with disabilities establishes the legal framework for a comprehensive social security system for PWD; Article 74 of the Act specifically states that the Government and Regional Governments are obliged to guarantee access for PWDs to water and sanitation services. Even so, Plan Indonesia, a national-scale non-governmental organisation (NGO), observed that PWDs cannot access regular sanitation and hygiene facilities despite a high desire to enhance their hygiene practices^16^.

Wilbur and Jones^17^ highlighted the urgency for Community-led Total Sanitation (CLTS), or called *sanitasi total berbasis masyarakat* STBM in Bahasa, the most widely adopted sanitation approach worldwide, including in Indonesia, being inclusive. Inclusiveness in the context of PWD means that PWD can perform their appraisal and sanitation profiles, or in other words, that they are involved in the sanitation-related decision-making process. However, PWDs are often not included in triggering discussions, have no voice in the community, and have their demands disregarded^18^. Therefore, to make CLTS more inclusive, the sanitation experiences of PWDs, their unique needs, and how they can be met must be determined.

This study uses mixed-method research to present an exploratory analysis of the perceptions of PWDs regarding sanitation within two districts of Eastern Indonesia. The main objectives of this study are threefold: (1) to assess the status of inclusive sanitation facilities of PWDs; (2) to understand knowledge, attitude, and practices (KAP) regarding sanitation for PWDs; (3) to identify barriers and to enable factors for PWDs to fully participate in sanitation decision making at household and community levels. In addition, the findings can be used to educate national and local governments about the importance of inclusive sanitation programs in improving the dignity, health, and quality of life of PWDs in Indonesia.

## Methods

### Study area

**Figure 1:**
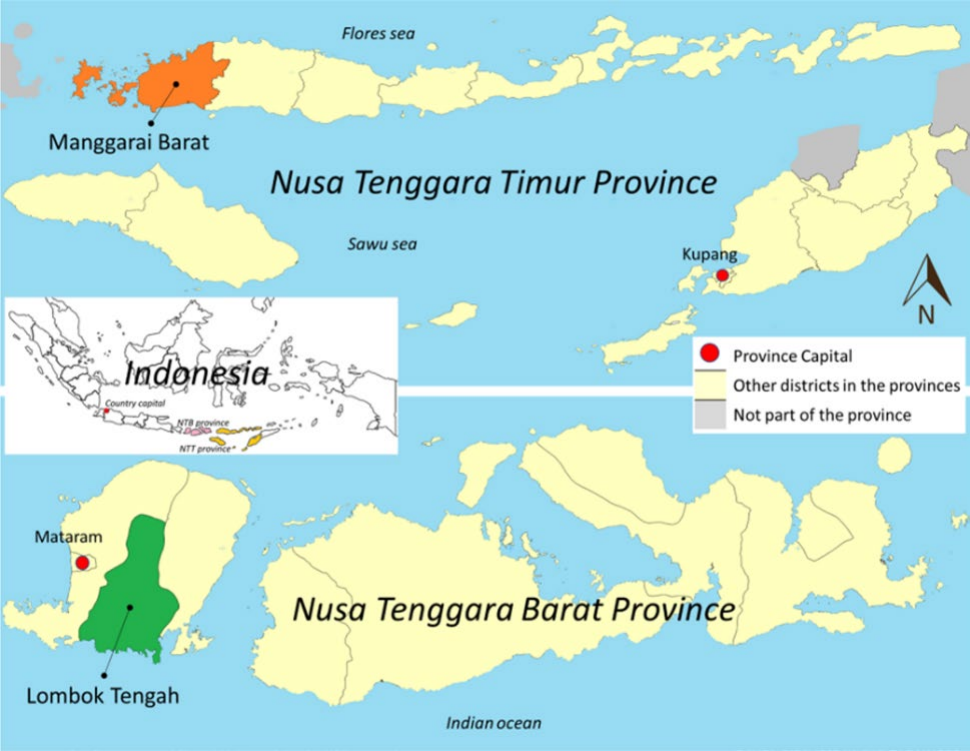
Map of study areas in NTT and NTB provinces. The map is drawn using ArcGIS ver. 10.8 (ESRI, Redlands, CA, USA).

This study was conducted in two districts in Nusa Tenggara Barat (Lombok Tengah) and Nusa Tenggara Timur (Manggarai Barat) in June 2022 (Fig. 1). The Central Bureau of Statistics (“BPS” in Bahasa) Nusa Tenggara Barat^19^ noted that access to improved sanitation in Lombok Tengah district in 2020 was 84.23%. Compared to data in 2019, 15.98% of the population still practice open defecation, while access to safely managed sanitation was only 1.74%^20^. In 2019, 68.24% of the population of Manggarai Barat owned private toilets, and 10.07% and 5.32% relied on shared and public facilities, respectively^21^. However, regarding safe sanitation, only roughly 36% have access to a septic tank or sewerage system; 61.88% of the total population used pits to dispose of faecal material, and the rest still practice open defecation in the pond/field/beach^22^. The number of PWDs in Lombok Tengah was 2848 in 2018^23^, whereas the number of PWDs in Manggarai Barat was 1145 in 2017^24^.

### Data collection

In this study, qualitative and quantitative methods were used. The qualitative data were obtained through in-depth interviews with relevant stakeholders and focus group discussions (FGD) with PWDs in Manggarai Barat and Lombok Tengah. Relevant stakeholders are: (1) representatives from the district’s agencies, e.g., social, health, and development planning agencies, (2) two NGOs, (3) village officers, and (4) the community health centre. In total, four FGDs were conducted, i.e., two FGDs in each location, and 24 in-depth interviews were conducted in two districts. The in-depth interviews last for 36 to 95 min. Meanwhile, the quantitative data were collected through a face-to-face questionnaire survey of 129 households with PWDs as family members in Lombok Tengah and Manggarai Barat (see Supplementary Material). The samples were randomly selected from the list of families with PWD members from 8 villages in Manggarai Barat and Lombok Tengah provided by the local disability organisations. Respondents were PWDs aged 15 years or older or the mother of PWD aged < 18 years who answered the questionnaire for the PWD. Informed consent was obtained from all respondents. The study protocol was approved by the Ethics Commission of Universitas Udayana Number 1448/UN14.2.2.VII.14/LT/2022. This research was performed in accordance with relevant guidelines/regulations, including the Declaration of Helsinki. Informed consent was obtained from all participants.

Quantitative data were collected using a pretested interviewer-administered questionnaire. If the PWD could not communicate directly with the interviewer, the companion helped with the communication. The questionnaire comprises seven sections: (a) respondent’s characteristics, (b) WASH conditions, (c) questions/statements related to knowledge, (d) questions/statements related to attitude, (e) economic conditions, (f) questions/statements related to practice, and (g) miscellaneous questions. The questionnaires are inspired by the KAP theory, often used to assess WASH-related behaviour^25,26^. The knowledge section comprises seven true–false statements related to the knowledge of WASH practices. The correct answers were added to produce a composite score representing the respondent's knowledge of WASH practices. The attitude section comprises five questions measuring the respondent’s attitude related to the participation of women, PWDs, and marginalised groups in WASH-related activities; and four questions about the respondent’s attitude related to WASH facilities at home. To assess the economic conditions, we asked the respondents about some items owned by the household member and observed house conditions. In addition, the questionnaire contains four questions related to perceptions of barriers or enabling factors related to the participation of women, PWDs, and marginalised groups in WASH-related activities at the household level. Lastly, the practice section comprises six questions about the participation level of PWDs in the WASH program in the community. The questionnaire can be found in the appendix. The survey assessed inclusive sanitation facilities based on six criteria: support tools or conditions for PWD to enter and use the toilet, door lock, privacy (no hole on wall or door to peek), lighting, soap availability, and closed trash bin.

### Data analysis

All interviews and FGDs were recorded with the consent of respondents. All interviews and FGDs were transcribed into Bahasa Indonesia. All transcripts were coded using content analysis. Data coding involves finding patterns in different categories and themes (Yu et al., 2009). Then, a basic causal loop diagram (CLD) was created based on the findings of qualitative interviews. CLD is a qualitative aspect of system thinking or system dynamics approach, which depicts the causal effect relationship among variables^27^. A growing interest exists in applying system thinking in the WASH sector^28^. CLD can help people with less technical knowledge to understand a complex system^29^. The basic CLD was then presented to relevant stakeholders in the reflective workshops conducted in both districts in early August 2022. They confirmed and gave suggestions on the CLD. The final CLD was then presented in this document.

All statistical analyses for quantitative data were conducted using IBM SPSS 25 (IBM Corp., Armonk, NY). Mean (m), standard deviation (SD), or frequency of quantitative variables were reported. Principal Component Analysis (PCA) was used to create composite variables for attitude, i.e., related to participation in the WASH programs, and practice, i.e., related to the participation level in the WASH programs. The PCA scores were obtained, in which higher scores represent a higher level of respondents’ attitudes and practices related to the WASH programs. PCA also was used to create the wealth index scores from the household’s assets and conditions, e.g., household ownership of TV, fridge, motorbike, conditions of house wall, floor, etc. The higher the first PCA component scores, the wealthier the respondent is^30,31^.

Several multivariate linear regressions were performed between the respondent's relevant socio-economic characteristics (SECs) as independent variables and knowledge, attitude, and practice as dependent variables, i.e., three regressions were conducted separately for each KAP. Another regression was also performed using the composite variable of knowledge and PCA scores of the attitude related to the participation in WASH-related activities as independent variables and PCA scores of the participation level of PWDs in the WASH program as a dependent variable. The final regression was between SECs, K, A, and P as independent variables and inclusive sanitation as the dependent variable.

## Results and discussion

### Descriptive statistics of quantitative survey

Some descriptive statistics of the respondents can be found in Table 1. The mean respondent’s age was 44.8 years (SD = 13.7). The mean years of respondent and household head education were 5.0 years (SD = 4.0) and 5.7 (SD = 4.1), respectively, which is equal to a primary school. The respondent’s role at home was mainly household wife (32.6%), followed by working household wife (20.9%), man household head (18.6%), children (10.9%), and other roles (17%). The primary disability type was physique (33.3%), followed by mute (27.1%) and paralysed body (12.4%).

**Table 1:** Descriptive statistics of respondents’ characteristics in the study areas.

Variable and categories	n	%
Location (n = 129)		
Lombok Tengah	113	87.6
Manggarai Barat	16	12.4
Gender (n = 129)		
Man	32	24.8
Woman	97	75.2
Using more than one water source (n = 129)	53	41.1
Water is available 24/7 (n = 129)	69	53.5
Main water source for the daily purpose (n = 129)		
Private tap	20	15.5
Public tap	14	10.9
Protected well	39	30.2
Unprotected well	43	33.3
Unprotected spring	5	3.9
River	3	2.3
Other	5	3.9
Main drinking water source (n = 129)		
Private tap	17	13.2
Public tap	20	15.5
Protected well	32	24.8
Unprotected well	28	21.7
Commercial potable water	23	17.8
Other	9	7
Time to collect water (n = 129)		
< 5 min (on premises)	83	64.3
5–15 min	28	21.7
16–30 min	15	11.6
31–45 min	3	2.3
Toilet ownership (n = 129)		
Own toilet	110	85.3
Public or shared toilet	15	11.6
Open defecation	4	3.1
Toilet classification according to JMP (n = 129)		
Safely managed	34	26.4
Basic	73	56.6
Limited	12	9.3
Unimproved	6	4.7
Open defecation	4	3.1
Disability types (n = 129)		
Blind	25	19.4
Deaf	10	7.8
Mute	35	27.1
Physique (hand or foot)	43	33.3
Paralysed body	16	12.4
Condition of toilet (n = 125)		
With lock	70	56.0
No hole to peek	64	51.2
With lamp	75	60.0
With water and soap	100	80.0
With bin	14	11.2
With support tools	7	5.6

The community mainly used the unprotected well for daily purposes and drinking water, followed by the protected well. Only 64.3% of all respondents had a water source on the premises, and 53.5% had a reliable 24/7 water supply. The male household head (54.3%) is the one who makes WASH decisions at home, followed by the mother (41.9%).

More than half of the respondents had a basic sanitation facility (56.6%), followed by safely managed (26.4%), limited access (9.3%), and improved access (4.7%), whereas 3.1% still practised open defecation. Regarding the completeness of the facility of the inclusive toilet (excluding open defecation), only four toilets (3.2%) met the standard of the inclusive toilet, i.e., have all six criteria for the inclusive toilet. The majority of the toilet had no support tools (94.5%) and bins (88.9%). Support tools here are the grab bar or tools that help disabled persons to sit and stand. Furthermore, only four-fifths of the toilets had water and soap, indicating a lack of toilet hygiene. Moreover, we acknowledge that determining whether a sanitation facility is inclusive will depend on the disability types. In the survey, we asked about the perceptions of respondents regarding the inclusivity of their WASH facilities (Table 2). Respondents said that the WASH facilities and conditions still do not meet the needs and conditions of PWDs, explained by the low score of the perception (0.4 out of 5).

**Table 2:** Descriptive statistics of respondents’ perceptions related to the WASH facility at home.

Questions/statements	Scale*	M (SD)
WASH facilities/infrastructure in this house are in accordance with the needs/conditions of women, persons with disabilities, and marginalised groups	1–5	2.6 (1.2)
The condition/condition of the WASH facilities/infrastructure at home is in accordance with the disability needs	0–1	0.4 (0.5)
It must be ensured that all family members can use the toilet and hand washing facilities easily	1–5	4.4 (0.7)
I cannot do other things I want because time is spent on WASH-related matters	1–5	2.6 (1.0)

*scale 1–5: 1 = very disagree, 5 = very agree; scale 0–1: 0 = no, 1 = yes; M = mean.

In Indonesia, the Regulation of the Ministry of Public Works and Human Settlement Number 14 the Year of 2017 provide detailed guidelines for an easily accessible toilet. The guideline includes the size and layout to facilitate wheelchair, provision of vertical and horizontal handrails on walls and doors, metal sheet on the bottom part of the door to facilitate opening the door by kicking and other general tools in the toilet such as mirror, waste bin, hand dryer, soap, jet shower, hand washing basin with appropriate elevation, etc. The regulation acknowledges that easily accessible toilets and buildings will support access for all (not only PWD). However, the provision of inclusive toilets seems less understood regarding benefits and thus has less priority among many stakeholders in Indonesia, including sanitation programs. Understanding the different needs of PWD concerning sanitation access will require discussion with PWD. However, even PWD have a limited understanding of their rights and how to meet their needs regarding sanitation access.

Table 3 shows the results of the questions or statements related to KAP. The knowledge related to WASH of PWD respondents was relatively low (M = 3.8). The mean of the composite scores of the attitude related to the participation level of PWDs is considered at the medium level, i.e., the mean was 2.3 from the maximum score of 4.1. Finally, the participation level of PWDs in the WASH-related program was low, i.e., the mean is 0.84 from the maximum score of 5.1.

**Table 3:** Descriptive statistics of respondents’ knowledge, attitude, and practice related to the participation of women, PWDs, and marginalised groups in WASH-related activities both at household and community levels.

Variables	Scale	M* (SD)
Knowledge related to sanitation or WASH		
Knowledge scores	0–7	3.8 (1.2)
Attitude		
In your opinion, how well is the involvement of women, PWDs, and marginalised groups in various WASH activities in your village?	1–5	2.2 (1.1)
Are women, PWDs, and marginalised groups invited to meetings in the village/community?	0–1	0.3 (0.4)
Women, PWDs, and marginalised groups should be involved in various WASH activities in this village	1–5	3.6 (1.1)
I feel comfortable attending village meetings	1–5	3.2 (1.3)
I feel comfortable expressing my opinion in village meetings	1–5	3.1 (1.3)
Composite scores of the attitude using PCA from 5 variables	0–4.1	2.3 (1.0)
Practices		
How involved are you in the preparation or PLANNING of WASH activities in your area?	1–5	1.4 (0.9)
How involved are you in the IMPLEMENTATION of WASH activities in your area?	1–5	1.4 (1.0)
How involved are you in MONITORING and EVALUATION of WASH activities in your area?	1–5	1.3 (0.8)
How much do you want to be involved in various WASH activities in your area?	1–5	3.5 (1.1)
I am rarely involved in WASH-related matters in my house	1–5	4.0 (0.9)
I actively monitor the involvement of women, the disabled, marginalised in various WASH activities in this area (eg., village/subdistrict)	1–5	2.0 (1.1)
Composite scores of the practice using PCA from 6 variables	0–5.1	0.84 (0.5)

*M = mean.

Furthermore, four statements are related to barriers or enabling factors: traditions, religion, economic conditions, and support from people. Table 4 shows that traditions and religious teaching were not barriers to participation in WASH programs, i.e., mean = 1.7, rather economic conditions as one of the barriers, i.e., mean = 3.3, as also found in another study^32^. Moreover, respondents felt that they obtained support from the people around them to participate in WASH-related activities.

**Table 4:** Respondents’ perceptions of barriers or enabling factors related to the participation of women, PWDs, and marginalised groups in WASH-related activities.

Questions/statements	Scale*	M (SD)
Traditions/customs in my village are one of the obstacles to the involvement of women, PWDs, and marginalised groups in various WASH activities	1–5	1.7 (0.8)
Religious teachings are one of the obstacles to the involvement of women, PWDs, and marginalised groups in various WASH activities	1–5	1.7 (1.1)
Economic conditions are one of the obstacles to my involvement in various WASH activities	1–5	3.3 (1.4)
People around me SUPPORT the involvement of women, PWDs, and marginalised groups in WASH-related activities/things	1–5	3.7 (0.9)

*1 = very disagree, 5 = very agree; M = mean.

### Regression analyses between SECs, KAP, and inclusive sanitation facilities

Regression analyses between SECs, KAP variables, and inclusive sanitation facilities are shown in Table 5. No significant variable was related to knowledge, whereas the household head’s education was significantly associated with the level of attitude (β = 0.26) and practice (β = 0.36). Furthermore, respondents with lower economic levels were more likely to participate in the WASH program, i.e., β =  − 0.23. The regression of all SECs, knowledge and attitude on practice revealed that the household head’s education, wealth index, and attitude were significantly related to the practice, i.e., β = 0.28, β =  − 0.22, β = 0.28. The attitude was positively associated with the practice, meaning that the higher the attitude of respondents toward participation in the WASH program, the more likely they were involved in the WASH program. Moreover, the regression of all SECs and KAP variables on inclusive sanitation facilities found that having a higher sanitation service level and a higher level of knowledge were associated with having an inclusive sanitation facility, i.e., β = 0.39 and β = 0.25, respectively. Finally, participation in WASH or sanitation program was not significantly related to having an inclusive sanitation facility at home.

**Table 5:** Regression analysis between socio-economic characteristics, knowledge, attitude, practice, and inclusive sanitation facility for the disabled (n = 129).

Independent variables	DV: knowledge			DV: attitude			DV: practice			DV: practice			DV: Inclusive sanitation facility for the disabled		
	B	SE B	β	B	SE B	β	B	SE B	β	B	SE B	β	B	SE B	β
Constant	4.79	0.79		2.16	0.61		0.46	0.59		− 0.17	0.67		− 2.18	1.10	
Gender	− 0.24	0.27	− 0.08	0.29	0.21	0.13	0.02	0.20	0.01	− 0.06	0.20	− 0.03	− 0.10	0.32	− 0.03
Age	− 0.01	0.01	− 0.13	− 0.01	0.01	− 0.13	0.01	0.01	0.09	0.01	0.01	0.13	0.01	0.01	0.09
Respondent’s education	0.00	0.04	0.00	− 0.02	0.03	− 0.06	− 0.01	0.03	− 0.04	− 0.01	0.03	− 0.03	− 0.01	0.05	− 0.02
Household head’s education	0.01	0.04	0.04	0.06	0.03	0.26*	0.09	0.03	0.36**	0.07	0.03	0.28*	0.07	0.05	0.17
Wealth index	0.01	0.12	0.01	− 0.04	0.09	− 0.04	− 0.23	0.09	− 0.23*	− 0.22	0.09	− 0.22*	0.01	0.15	0.01
Sanitation facility ladder (service level)	− 0.08	0.13	− 0.06	0.01	0.10	0.01	− 0.09	0.10	− 0.09	− 0.10	0.09	− 0.09	0.70	0.15	0.39***
Knowledge										0.00	0.07	0.00	0.33	0.11	0.25**
Attitude										0.28	0.08	0.28**	− 0.02	0.14	− 0.01
Practice													0.041	0.149	0.04
R^2^	0.03			0.10			0.16			0.23			0.23		

Blank cell means that the independent variable(s) is not used in that regression.

DV,  dependent variable.

*p ≤ 0.05; **p < 0.01; ***p < 0.001.

### CLD of factors related to the participation of PWDs in the sanitation program

**Figure 2:**
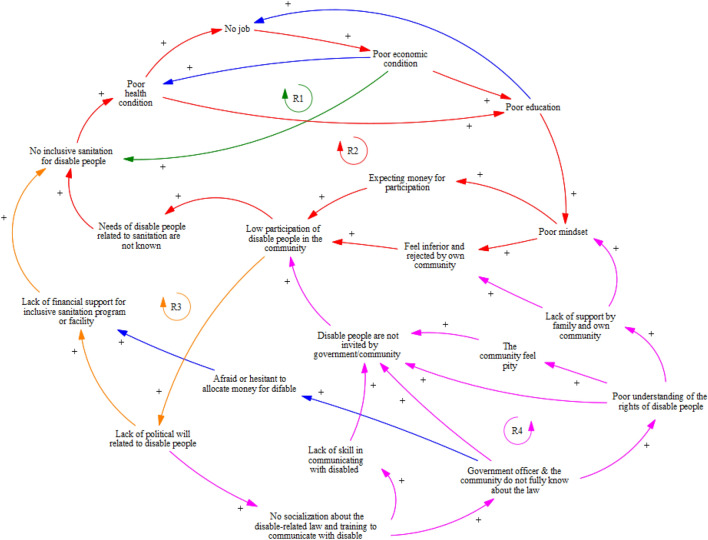
Causal loop diagram of factors related to the participation of disabled people in sanitation program: A case study from Lombok Tengah Regency.

**Figure 3:**
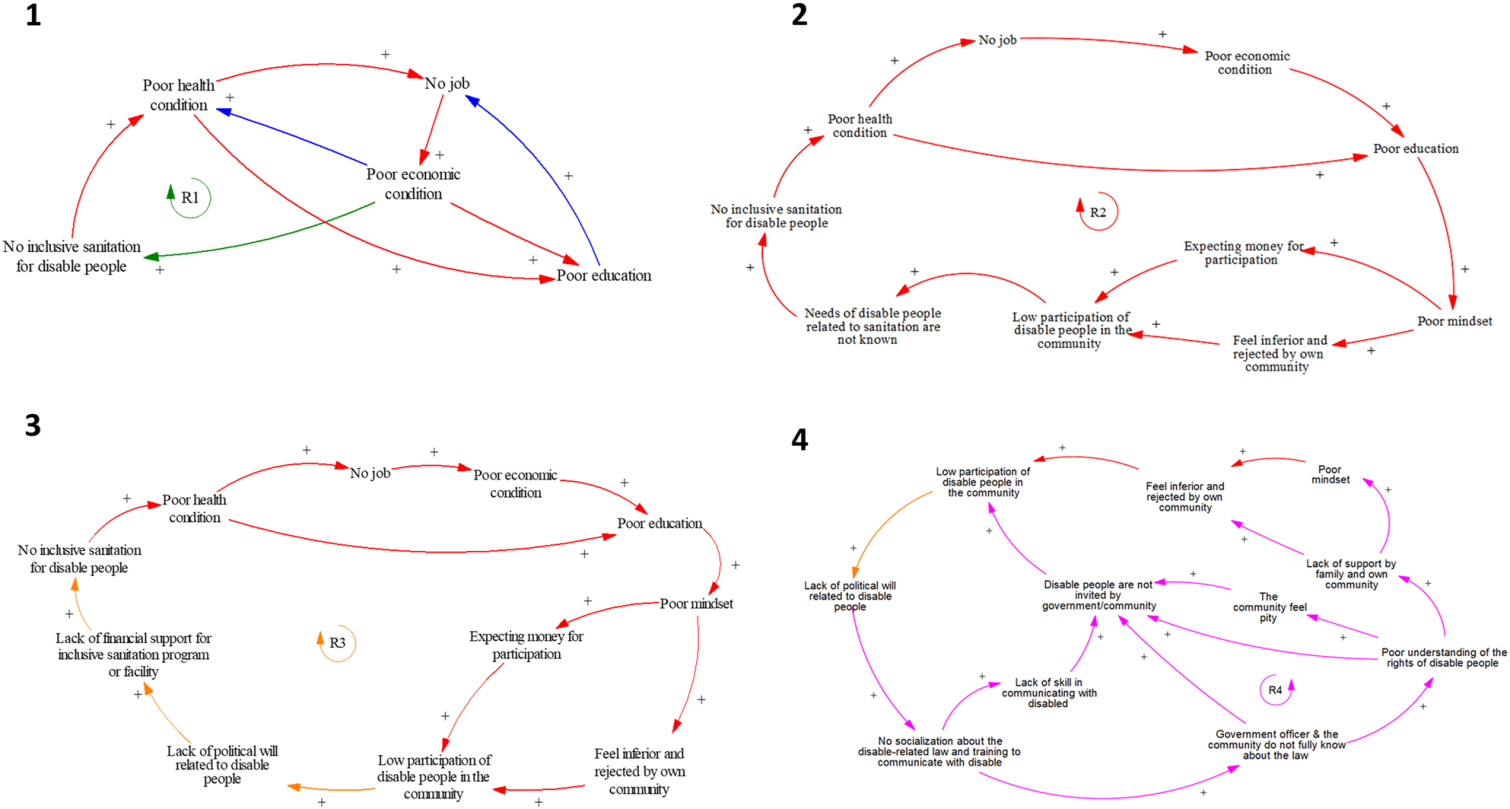
Four main causal loop diagram sub-models in the system related to the participation of disabled people in sanitation programs.

The CLD based on in-depth interviews with relevant stakeholders in Lombok Tengah is shown in Fig. 2. Four main subsystems are considered important in understanding the participation level of PWDs in the sanitation program and its relation to having inclusive sanitation facilities at home and other factors (Fig. 3).

Sub-model 1 shows interconnections between inclusive sanitation facilities for PWDs and long-term consequences, i.e., poor economic, health, and education conditions. Furthermore, loop R1, based on the qualitative interviews, confirms the argument of the bi-directional relationship between disability and poor economic condition by Pinilla-Roncancio^33^. Non-inclusive sanitation facilities may result in poor health conditions for PWDs. Five FGD participants said they encountered an accident due to poor sanitation facilities. Poor health conditions make it challenging for PWDs to find a proper job^34,35^ and obtain education^36^. All these conditions worsen their economic condition, which can lead to non-inclusive sanitation at home.

“There is a hole in the toilet’s floor and she often falls due to it”—FGD with PWDs, female, Lombok Tengah.

Sub-model 2 depicts internal barrier factors for the participation of PWDs in sanitation programs, i.e., barriers that come from the person itself, e.g., poor mindset and expecting money for participating in any event, including sanitation-related events or programs. These internal barriers can be influenced by poor education gained by PWDs, which is a long consequence of poor economic and health conditions due to having no inclusive sanitation access. In addition, low participation of PWDs in any community event or meeting causes the community or government officer to be unaware of their inclusive sanitation needs, which is supported by other studies outside WASH settings^37^.

Given that most of the PWDs in the study areas come from low-middle income households, the quantitative survey indicates that economic condition is one of the barriers to participation in sanitation programs (see our explanation about Table 4). This condition creates the expectation that PWDs will receive monetary compensation for participating in activities, which cannot always be provided by the event organiser, e.g., the village office or any district agency.

“... Well, that is the obstacle for the headman because when the headman wants to do a program, of course, he will invite the elders of the disabled in the area while the orientation of the disabled is the contents of this envelope (money)...”—in-depth interview, disability NGO, male, Lombok Tengah.

The sub-model 3 indicates a long-term causal relationship between political will, absence of inclusive sanitation, and low participation of PWD. The lack of political will at the regency, district, or village levels results in a lack of financial support for sanitation programs, including the provision of inclusive sanitation facilities for PWDs. Two in-depth interview respondents stated that the declaration of 100% open defecation free at the regency level significantly reduces the fund for sanitation programs, even though they know that some households still do not have access to toilets, i.e., those households use the public or shared toilet. As a result, subsidies or support for making inclusive sanitation facilities for PWDs is limited, even though many PWDs are poor. Furthermore, low participation of PWDs in community events or meetings may cause a lack of political will regarding the needs and rights of PWDs. PWDs attending a community event or meeting can raise their voices and pressure political leaders and government officials, improving the social inclusion of PWDs^38^.

“…. A problem because in Puyung village there are 600 people who don’t have latrine while the funds are only 400 million, they said they didn’t cover it. Back again to the mentality of the Headman who always does activities without making innovations...”—in-depth interview, disability NGO, male, Lombok Tengah.

“… we have declared open defecation free (ODF) in the whole district, so we assume that there is no open defecation anymore although not all people have their own toilet. What we mean here is accessibility (everyone can access the toilet). People may not have a toilet, but they can use the shared one.”—in-depth interview, district health agency, female, Lombok Tengah.

“…. They want to say something (disabilities), but sometimes they are embarrassed so that when meeting with the governments they become weak and do not dare to express opinions…”—in-depth interview, disability NGO, male, Lombok Tengah.

In contrast to sub-model 2, sub-model 4 portrays external barrier factors, i.e., barriers that come from outside the person, for the participation of PWDs in sanitation programs. These external factors influence the internal barrier factors, e.g., poor mindset and the feeling of inferiority and rejection by the community, which are caused by the external factor of a lack of support from family and/or one’s community. Familial support is widely recognised as critical for PWD participation^39^. Interestingly, one of our respondents said that PWDs may be ignored or rejected by their own community or village but feel accepted by other communities. That is because PWD’s own family or neighbour often underrate PWD. Moreover, many people still think that PWDs are incapable of participating in various activities, i.e., feelings of pity or sympathy^40^, which is one of the reasons why PWDs are not invited to the WASH program or community activities.

“… my friends with disabilities rarely go out because of feeling inferior”—in-depth interview, disability NGO, male, Lombok Tengah.

“one of the obstacles for disabled people in Lombok is they are more successful and more (feeling) accepted outside of their village, (but) not in their village… because if he/she goes to other villages, what they see (the community in other villages) is what they are doing now and they become a motivation for those people (in other villages outside their own village)”—in-depth interview, disability NGO, male, Lombok Tengah.

“… there is something about the mindset of the village officers who feel sorry (for PWD). We feel sorry for inviting him/her (PWD)… but again, the chairman of X (mentioning one stakeholder) says that ‘I feel sorry (for inviting PWD)’ …”—in-depth interview, disability NGO, male, Lombok Tengah.

According to sub-model 4, the root cause of this problem is a lack of political will on the part of the political or governmental leader. A lack of political will was also cited as a reason for a lack of legal socialisation, which resulted in a poor understanding of the rights of PWDs by government officials and the community, either at the village or regency levels. The main consequence of this situation is that PWDs are not invited to community meetings, e.g., village meetings, which limits their participation in the community and sanitation programs. Furthermore, a lack of political will may result in the lack of training to communicate well with PWDs, e.g., how to obtain their feedback on a specific topic and how to facilitate PWDs to share their thoughts in a community meeting, as mentioned by participants in the reflective workshop. As a result, government bodies are reluctant to invite PWDs to community meetings, or if PWDs are invited, they rarely give their opinions regarding the topic. One of the reasons is the PWD's poor mindset (see the previous explanation about sub-model 2).

“If they (village or government officers) read the law, they will understand that there is a right of the (disable) people that is not fulfilled… none of the village officers know anything related to the law (regarding disabled people).”—in-depth interview, disability NGO, male, Lombok Tengah.

The legal situation concerning PWDs in Indonesia facilitates the participation of PWDs in any activity. For example, The Village Law Number 6 in 2014 indicates that all people, including PWDs, have the right to participate in village activities. Moreover, the national government approved Law Number 8 in 2016 regarding PWDs, emphasising the rights of PWD, e.g., participate in any activities, have inclusive access to a public facility, and obtain an inclusive education facility. The local government is also required to facilitate and involve PWDs in cultural activities, i.e., one activity that can enhance PWD participation in their community. Moreover, Chapter 100 in Law Number 8 states, "The government and local governments can facilitate the provision of easily accessible facilities in single-dwelling houses inhabited by persons with disabilities.” Therefore, even though the local government is not required to provide accessible facilities, such as toilets, there seems to be an opportunity for the local government to allocate funds to build inclusive sanitation at PWD homes. Therefore, legislation can act as a supportive tool to facilitate PWD participation^32^, especially in the Indonesian context.

“…. I only joined the program from Lombok Independent Disabilitas Indonesia (LIDI) (an NGO for PWD). In addition, I have never received an invitation, and this is the first time I have been invited to a discussion (Research FGD)…”—FGD with PWDs, male, Lombok Tengah.

“…. There was never a call or invitation from the village. Everything else is determined by the government… ”—FGD with PWDs, male, Lombok Tengah.

### Study implications

The findings of this study highlight various structural and social barriers to meeting the inclusive sanitation facility in their homes. These are consistent with previous studies where, around the world, structural barriers include steps or raised latrines, small latrines, squatting toilets, lack of support bars, inaccessible sinks, and slippery or uneven paths, often preventing PWDs from using sanitation facilities comfortably^41^. Meanwhile, social barriers revolve around negative stigma towards PWDs, inadequate autonomy, privacy, and dignity when using sanitation facilities^8,41^. This issue, at the moment, needs to be captured by the SDGs metric, hence leading to more attention towards meeting the sanitation need of the PWDs. Therefore it is crucial to measure individual-level sanitation access by including inclusivity criteria to understand better sanitation challenges among vulnerable individuals, such as PWDs (for example, see Quality of WASH Access questionnaire^42^).

We consider that one of the key root causes of the low participation of PWDs is the misunderstanding of the rights of PWDs and the lack of capacity of government staff to work and communicate with PWDs. The Convention on the Rights of Persons with Disabilities has been ratified by almost all countries in the world^43^. However, our findings indicate that this national-level initiative is not always transferred to a lower or local level, as found in other contexts^6^. In the Indonesian context, a national-level policy will be easier to implement if there is an implementing or local regulation at the lower level, e.g. at the provincial or district levels. Therefore, the government body at a lower level, e.g., district or subdistrict level, should develop a local regulation that supports the implementation of the national laws. However, this can occur if a strong political will exists to involve vulnerable groups, such as PWDs, in the activities^44^.

In addition, efforts must be directed toward the socialisation of PWD law, as mentioned previously in sub-model 4, to the district and community level, e.g., district office, village officers, and PWD families. This would hopefully change the negative stigma toward PWDs, increase support from their families and communities, and enhance awareness of the potential capacity of PWDs. Furthermore, given that the statistical analysis found that high education attainment of the household head is positively associated with the level of attitude and participation of PWDs, socialisation can target highly educated household heads as the main participants. Resultantly, they can be a role model for other household heads.

The socialisation of PWD law will also affect the knowledge of the PWD themselves about their rights, e.g., attending and giving opinions in the community meeting. Furthermore, as suggested by the regression analysis, it is necessary to improve the knowledge of PWD themselves related to sanitation and hygiene, e.g., what sanitation facility they should have at home, the PWD law that supports their rights, etc. This may increase their willingness to participate in the community meeting. The activity to improve PWD knowledge can also be conducted by the PWD associations.

Capacity building of relevant government bodies and stakeholders is necessary, so they can effectively involve and gather the opinions of PWDs, either in small-scale discussions or community meetings, as also noticed in other studies^6,45^. For example, the meeting’s moderator should be directed to allocate time for PWDs to express their opinions in the community meeting, or there should be training on creating a discussion situation where PWDs can express their opinions conveniently. Special training is also needed to communicate and gather information from the deaf, blind, and mute PWDs in the discussion. The local PWDs organisations can lead the training series targeting village and district officials.

A higher service level of sanitation facilities is associated with a more inclusive facility. However, the local government must continue ensuring PWDs have access to basic sanitation services. According to the data, almost 20% of our respondents had limited, unimproved, or even still practiced open defecation. Having an inclusive toilet as per PWD needs can prevent PWDs from getting into accidents while using it. Furthermore, the construction of the latrine should meet the needs and conditions of the PWDs at home^46^.

Moreover, the presence of associations for PWDs can create pressure on political leaders to support the involvement of PWDs in sanitation programs, besides pushing the local government to implement the PWD law^47^. In Lombok Tengah, for example, LIDI Foundation, a local non-governmental organisation based in Lombok that focuses on PWD empowerment, has helped to promote inclusive sanitation in the area. They engage PWDs to produce a latrine that fits the needs of PWDs. As a result, the NGO received an award at the national level related to sanitation in 2022. This national-level recognition indirectly raises the attention discussion toward PWD in that area.

### Study limitations and recommendations for future studies

The quantitative survey and qualitative interviews were conducted concurrently, making it difficult to confirm some quantitative findings, e.g., how household head education influences the level of PWD participation. We also think that more respondents from various locations are needed to obtain e better picture of the situation of PWDs in Indonesia. Future studies should investigate the influence of leadership or political systems on the provision of inclusive sanitation access for PWD. Furthermore, 26.4% of safely managed sanitation services among PWD respondents should be interpreted with caution. According to the data, the level of safely managed sanitation services in Indonesia was 7.64%^48^. Thus, the 26.4% obtained in our survey raises a question. That could be because the respondents are over-claimed that their septic tank is regularly emptied which could not be confirmed at that moment. In the Indonesian context, a household septic tank that is regularly emptied, e.g., every three to five years, indicates a safely managed sanitation facility^49^. A closer inspection should be conducted to confirm this finding. Furthermore, our study scope is only for physical disabilities. We suggest studying further other types of disability, e.g., intellectual or mental health disabilities. Moreover, the toilet design requirements for each type of PWD vary, thus, future studies should consider this in their research to give a more accurate assessment of the inclusiveness of sanitation facilities in PWD houses.

## Conclusion

The SDG “ aim to "Leave no one” behind," emphasising the importance of including PWDs in WASH and respecting their fundamental rights. Our study reveals that PWDs in Indonesia, especially in rural areas and those who are economically weak, face various challenges to meet the inclusive sanitation facility in their homes. Improving the general knowledge of PWD themselves, as implied by the regression analysis, related to sanitation and hygiene may result in more inclusive sanitation access at home. Even though the participation of PWD in sanitation programs is not significantly related to inclusive sanitation access, qualitative interviews reveal the opposite, i.e., participation remains critical for inclusive sanitation access. The quantitative survey indicates that financial condition is one of the barriers to participation. Qualitative analysis reveals that misunderstanding of the rights and needs of PWDs and the limited capacity of government staff in facilitating and communicating with PWDs are key barriers to participation, even though the Indonesian laws favour PWD participation and fulfilment of PWD needs, including inclusive sanitation access. This study also suggests that the barriers can come from different levels, e.g., the household, village, to the district level, and/or different actors, e.g., family, the community, village officers, district officers, and the PWD itself. Thus, the involvement of all actors, including associations for PWDs or NGOs, at all levels is necessary to provide inclusive sanitation access for PWD or marginalised groups, e.g., by pushing the creation and/or implementation of implementing regulation at the local level. Finally, the results of this study can be used as a basis for improving PWD participation in sanitation programs in Indonesia, mainly because scientific research or evidence on this topic is rare in Indonesia. We wish that this paper can draw more attention from the national and local governments to the provision of inclusive sanitation access for PWD, not only in Indonesia but also in other low and middle-income countries.

## Supplementary Information

Supplementary Information.

## Data Availability

The datasets generated and analysed during this study are not publicly available because the disability issue is considered sensitive in Indonesia but are available from the corresponding author on reasonable request.

## References

[1] Mara D, Lane J, Scott B, Trouba D (2010). Sanitation and health. PLoS Med..

[2] WHO & UNICEF. Progress on household drinking water, sanitation and hygiene 2000–2020: five years into the SDGs. *Joint Water Supply, & Sanitation Monitoring Programme* 1–164 (2021).

[3] United Nations General Assembly. *Transforming Our World: The 2030 Agenda for Sustainable Development*. https://sustainabledevelopment.un.org/content/documents/7891Transforming%20Our%20World.pdf (2015) 10.1007/s13398-014-0173-7.2.

[4] United Nations. Convention on the rights of persons with disabilities. https://www.ohchr.org/en/instruments-mechanisms/instruments/convention-rights-persons-disabilities (2006). 10.1515/9783110208856.203 18348362

[5] WHO. https://www.who.int/news-room/fact-sheets/detail/disability-and-health.

[6] Scherer N (2021). The inclusion of rights of people with disabilities and women and girls in water, sanitation, and hygiene policy documents and programs of Bangladesh and cambodia: Content analysis using equiframe. Int. J. Environ. Res. Public Health.

[7] Mactaggart I (2018). Access to water and sanitation among people with disabilities: Results from cross-sectional surveys in Bangladesh, Cameroon, India and Malawi. BMJ Open.

[8] Banks LM (2019). Are current approaches for measuring access to clean water and sanitation inclusive of people with disabilities? Comparison of individual- and household-level access between people with and without disabilities in the Tanahun district of Nepal. PLoS ONE.

[9] Dwipayanti NMU (2022). Inclusive WASH and sustainable tourism in Labuan Bajo, Indonesia: Needs and opportunities. J. Water Sanit. Hyg. Dev..

[10] Groce N, Kett M, Lang R, Trani J-F (2011). Disability and poverty: The need for a more nuanced understanding of implications for development policy and practice. Third World Q..

[11] Dibley T, Tsaputra A (2019). Changing laws, changing attitudes: the place of people with disability in Indonesia. Contentious Belonging The Place of Minorities in Indonesia.

[12] WHO & The World Bank. *World Report on Disability*. https://www.who.int/publications/i/item/9789241564182 (2011).

[13] Kusumastuti P, Pradanasari R, Ratnawati A (2014). The problems of people with disability in Indonesia and what is being learned from the world report on disability. Am. J. Phys. Med. Rehabil..

[14] Larasati, D., Huda, K., Cote, A., Rahayu, S. K. & Siyaranamual, M. Policy brief: Inclusive social protection for persons with disability in Indonesia. https://www.tnp2k.go.id/downloads/inclusive-social-protection-for-persons-with-disability-in-indonesia (2019).

[15] Suharto S, Kuipers P, Dorsett P (2016). Disability terminology and the emergence of ‘diffability’ in Indonesia. Disabil. Soc..

[16] Triwahyudi, W. & Setiawan, E. Disability Inclusion in WASH: What has been achieved and how can this help other practitioners? In *37th WEDC International Conference* (WEDC International Conference, 2014).

[17] Wilbur, J. & Jones, H. *Disability: Making CLTS Fully Inclusive*. *Frontiers of CLTS: Innovations and Insights 3.* https://sanitationlearninghub.org/resource/disability-making-clts-fully-inclusive/ (2014).

[18] Jones, H., Parker, K. J. & Reed, R. Water supply and sanitation access and use by physically disabled people: A literature review. WEDC, Loughborough University. https://asksource.info/sites/all/modules/pubdlcnt/pubdlcnt.php?fid=5148 (2002).

[19] BPS Nusa Tenggara Barat. Persentase Ruta yang Memiliki Akses Terhadap Layanan Sanitasi Layak (Persen), 2018–2020. https://ntb.bps.go.id/indicator/29/336/1/persentase-ruta-yang-memiliki-akses-terhadap-layanan-sanitasi-layak.html (2021).

[20] POKJA AMPL-BM Provinsi NTB. *Roadmap BASNO Menuju Sanitasi Aman Nusa Tenggara Barat 2020–2023*. (2020).

[21] BPS Manggarai Barat. Persentase Rumah Tangga menurut Penggunaan Fasilitas Buang Air Besar (Persen), 2017–2019. https://manggaraibaratkab.bps.go.id/indicator/29/96/1/persentase-rumah-tangga-menurut-penggunaan-fasilitas-buang-air-besar.html (2020).

[22] BPS Manggarai Barat. Persentase Rumah Tangga menurut Jenis Tempat Pembuangan Akhir Tinja (Persen), 2015–2017. https://manggaraibaratkab.bps.go.id/indicator/29/99/1/persentase-rumah-tangga-menurut-jenis-tempat-pembuangan-akhir-tinja.html (2020).

[23] Dinas Sosial. Detail jumlah penyandang disabilitas menurut kecamatan di kabupaten lombok tengah tahun 2018. https://sektoral.lomboktengahkab.go.id/detail-data-sektoral/5d706508ebd.

[24] Statistics of Nusa Tenggara Timur Province. Jumlah Penyandang Masalah Kesejateraan Sosial (PMKS) (Jiwa), 2017. https://ntt.bps.go.id/indicator/27/586/1/jumlah-penyandang-masalah-kesejateraan-sosial-pmks-.html.

[25] Sibiya JE, Gumbo JR (2013). Knowledge, attitude and practices (KAP) survey on water, sanitation and hygiene in selected schools in Vhembe District, Limpopo, South Africa. Int. J. Environ. Res. Public Health.

[26] Lampard-Scotford AR (2022). Knowledge, attitudes, practices and behaviours (KAPB) around water, sanitation and hygiene (WASH) in villagers exposed to schistosomiasis in Zimbabwe. PLOS Water.

[27] Peters DH (2014). The application of systems thinking in health: Why use systems thinking ?. Heal. Res. Policy Syst..

[28] Valcourt N, Javernick-will A, Walters J, Linden K (2020). System approaches to water, sanitation, and hygiene: A systematic literature review. Int. J. Environ. Res. Public Health.

[29] Daniel D (2021). A system dynamics model of the community-based rural drinking water supply program ( PAMSIMAS ) in Indonesia. Water.

[30] Daniel D (2021). Contextual determinants of general household hygiene conditions in rural Indonesia. Int. J. Environ. Res. Public Health.

[31] Houweling TAJ, Kunst AE, Mackenbach JP (2003). Measuring health inequality among children in developing countries: Does the choice of the indicator of economic status matter?. Int. J. Equity Health.

[32] Hästbacka E, Nygård M, Nyqvist F (2016). Barriers and facilitators to societal participation of people with disabilities: A scoping review of studies concerning European countries. Alter.

[33] Pinilla-Roncancio M (2015). Disability and poverty: Two related conditions. A review of the literature. Rev. Fac. Med..

[34] Bonaccio S, Connelly CE, Gellatly IR, Jetha A, Martin Ginis KA (2020). The participation of people with disabilities in the workplace across the employment cycle: Employer concerns and research evidence. J. Bus. Psychol..

[35] Jurado-Caraballo MÁ, Quintana-García C, Rodríguez-Fernández M (2020). Trends and opportunities in research on disability and work: An interdisciplinary perspective. BRQ Bus. Res. Q..

[36] Wibowo SB, Muin JA (2018). Inclusive education in Indonesia: Equality education access for disabilities. KnE Soc. Sci..

[37] Franklin A, Sloper P (2006). Participation of disabled children and young people in decision making within social services departments: A survey of current and recent activities in England. Br. J. Soc. Work.

[38] Ahmad S (2022). The influence of decision making on social inclusion of persons with disabilities: A case study of Khyber Pakhtunkhwa. Int. J. Environ. Res. Public Health.

[39] Rohwerder, B. *Disability stigma in developing countries*. *K4D Helpdesk Report* https://assets.publishing.service.gov.uk/media/5b18fe3240f0b634aec30791/Disability_stigma_in_developing_countries.pdf (2018).

[40] Sharma N, Pratap Yadav V, Sharma A (2021). Attitudes and empathy of youth towards physically disabled persons. Heliyon.

[41] Groce N, Bailey N, Lang R, Trani JF, Kett M (2011). Water and sanitation issues for persons with disabilities in low- and middle-income countries: A literature review and discussion of implications for global health and international development. J. Water Health.

[42] White S, Kuper H, Itimu-Phiri A, Holm R, Biran A (2016). A qualitative study of barriers to accessing water, sanitation and hygiene for disabled people in Malawi. PLoS ONE.

[43] United Nations. Convention on the Rights of Persons with Disabilities (CRPD). *United Nations* https://treaties.un.org/Pages/ViewDetails.aspx?src=TREATY&mtdsg_no=IV-15&chapter=4&clang=_en (2023).

[44] Tsekleves E (2022). Community engagement in water, sanitation and hygiene in sub-Saharan Africa: Does it WASH?. J. Water Sanit. Hyg. Dev..

[45] Jones, N., Presler-Marshall, E. & Stavropoulou, M. *Adolescents with Disabilities: Enhancing Resilience and Delivering Inclusive Development*. https://odi.org/en/publications/adolescents-with-disabilities-enhancing-resilience-and-delivering-inclusive-development/ (2018).

[46] Othman Z, Buys L (2016). Towards more culturally inclusive domestic toilet facilities in Australia. Front. Archit. Res..

[47] Acharya S (2016). Disability Law Implementation: The Role of NGOs and INGOs in Nepal. SSRN Electron. J..

[48] POKJA PPAS. Akses Sanitasi Aman Tahun 2017–2020 di Indonesia. *Nawasis.org* 1 https://www.nawasis.org/portal/galeri/read/akses-sanitasi-aman-tahun-2017-2020-di-indonesia/52219 (2021).

[49] Odagiri M (2021). Safely managed on-site sanitation: A national assessment of sanitation services and potential fecal exposure in Indonesia. Int. J. Environ. Res. Public Health.

