# Supplementary material for: Access to inclusive sanitation and participation in sanitation programs for people with disabilities in Indonesia
Source: Sci Rep. 2023 Mar 15;13:4310. doi: 10.1038/s41598-023-30586-z (PMC10017721; doi:10.1038/s41598-023-30586-z)
Supplement: Supplementary file 1 — Supplementary Information. [file 41598_2023_30586_MOESM1_ESM.docx]

Supplementary material

**Access to inclusive sanitation and participation in sanitation programs for people with disabilities in Indonesia**

D. Daniel ^1^, Anindrya Nastiti ^2^, Hana Yesica Surbakti ^3^, Ni Made Utami Dwipayanti ^4^

^1^ Department of Health Behavior, Environment, and Social Medicine, Faculty of Medicine, Public Health and Nursing, Universitas Gadjah Mada, Yogyakarta, Indonesia

^2^ Environmental Management Technology Research Group, Faculty of Civil and Environmental Engineering, Bandung Institute of Technology, Bandung, Indonesia

^3^ Department of Public Health, The Graduate School, Universitas Gadjah Mada, Yogyakarta, Indonesia

^4^ School of Public Health, Udayana University, Denpasar, Indonesia

Corresponding: anindrya@tl.itb.ac.id

# Quantitative Survey Instrument

|  | **A - Household Information** |
| --- | --- |
| 1 | Name of interviewee/respondent |
| 2 | Name of household head |
| 3 | What is the gender of the respondent? |
| 4 | What is your role or position in this house? |
| 5 | How old is the respondent? |
| 6 | What grade was your last formal education? |
| 7 | What grade was the last formal education of the head of the family? |
| 8 | What beliefs do you follow? |
| 9 | Who makes decisions at home regarding financial expenditures related to water, hygiene and sanitation? |
|  | **B - WASH Condition** |
| 1 | Where do you get your water for your daily needs? |
| 2 | Which is your main source of drinking water? |
| 3 | Is the water always available or available 24 hours (not off and on)? |
| 4 | How long does it take (minutes) to fetch water from the main water source, wait in line, fill the container and return (travel and return)? |
| 5 | Where do you usually defecate? |
| 6 | If you defecate in the toilet (whether it's your own or not), what type of toilet is it? |
| 7 | If you defecate in the toilet (whether it's your own or not), where is the waste disposed of? |
| 8 | Who takes care of the most WASH-related matters at home (such as fetching water)? |
| 9 | Are there persons with disabilities in this house? |
| 10 | If yes, what is the form of disability? |
| 11 | If there is a disability, is the toilet inclusive? |
| 12 | if there is a toilet, please take a picture with a cellphone camera |
|  | **C - Knowledge** |
| 1 | There are government regulations/directions regarding the involvement of women, people with disabilities, and marginalized groups in various WASH programs in Indonesia |
| 2 | Women have a greater chance of getting diarrhea than men |
| 3 | Women and children are a vulnerable group if adequate WASH facilities are not provided for them |
| 4 | Cleaning and sanitation matters are women's business only |
| 5 | Women have the right to have adequate WASH facilities |
| 6 | Did you know that septic tanks must be vacuumed at least once every 5 years |
| 7 | Women tend to use more water than men |
|  | **D - Attitude** |
| 1 | In your opinion, how well are women, persons with disabilities, and marginalized groups involved in various WASH activities in your area? |
| 2 | Traditions/customs in my area are one of the obstacles to the involvement of women, persons with disabilities, and marginalized groups in various WASH activities |
| 3 | Religious teachings are an obstacle to the involvement of women, persons with disabilities, and marginalized groups in various WASH activities |
| 4 | Economic conditions are one of the one barrier to my involvement in various WASH activities |
| 5 | facilities/infrastructure in this area are in accordance with the needs/conditions of women, persons with disabilities, and marginalized groups |
| 6 | If there are persons with disabilities at home, the condition/condition of WASH facilities/infrastructure at home is in accordance with the needs of persons with disabilities |
| 7 | If there are women and children in the house, the condition/condition of the WASH facilities/infrastructure in my house is in accordance with the needs and safe for women and children |
| 8 | If there are no toilets or WASH facilities at home, I feel uncomfortable using/ accessing WASH facilities outside my home (such as public toilets, faucets or communal wells, etc.). |
| 9 | There should be a balanced division of roles between men and women in water and sanitation matters at home |
| 10 | I can't do other things I want because time is spent on WASH-related matters |
| 11 | I want my time for business about WASH is reduced so that I can do other things |
| 12 | Are women, persons with disabilities, and marginalized groups invited to meetings in the village/community? |
| 13 | If invited, are the opinions of women, persons with disabilities, and marginalized groups heard/respected? |
| 14 | If invited, can women, persons with disabilities, and marginalized groups actively participate in opinion and decision-making processes? |
| 15 | People around me support the involvement of women, persons with disabilities, and marginalized groups in WASH-related activities/things |
| 16 | I feel comfortable attending village meetings |
| 17 | I feel comfortable expressing opinions in village meetings |
| 18 | Women, persons with disabilities, and marginalized groups should be involved in various WASH activities in the region |
| 19 | Women should have the same opportunities as men in making decisions related to WASH at home |
| 20 | Women should have the same opportunities as men in leadership around WASH in the village |
| 21 | important to ensure that all family members can use the toilet and hand washing facilities easily |
|  | **E - Economic conditions** |
| 1 | Approximately, how much do you spend in a month? |
| 2 | What wall materials does your house have? |
| 3 | What type of roof does your house have? |
| 4 | What type of flooring does your house have? |
| 5 | Are any of these items owned by a member of your family who lives in this house (and is also undamaged)? |
|  | **F - Practice** |
| 1 | How involved are you in the preparation or planning of WASH activities in your area? |
| 2 | How involved are you in implementing WASH activities in your area? |
| 3 | How involved are you in monitoring and evaluating WASH activities in your area? |
| 4 | How much do you want to be involved in various WASH activities in your area? |
| 5 | In your opinion, which activities require your involvement (women, persons with disabilities, and marginalized groups) in WASH activities? |
| 6 | Of the several activities you mentioned, which activities require the involvement of women, persons with disabilities, and marginalized groups the most? |
| 7 | I am rarely involved in WASH-related matters in my home |
| 8 | Men like to take care of WASH-related matters at home |
| 9 | I actively monitor the involvement of women, disabled, marginalized in various WASH activities in this area |
|  | **G - Others** |
| 1 | I have been injured because of things related to WASH |
| 2 | I have been harassed (physically, mentally, sexually) because of things related to WASH |
| 3 | I have sacrificed important activities for things related to WASH |
| 4 | At times I am sick or unavailable, men take care of WASH-related matters at home |
| 5 | approx, how long do you talk to your neighbors about your life in one day? (in hours) |
| 6 | How often do you talk to your neighbors about water, hygiene and sanitation? |
| 7 | How often do you watch TV in a day? |
| 8 | How often do you open your social media in a day (eg facebook, IG, tiktok, etc.)? |
| 9 | Take the GPS location of the house |
| 10 | Write down if there is a note about this respondent |

# Qualitative Research Instrument

**Guide to Focus Group Discussions (Disability)**

*Consent*

Thank you for being willing to participate. We are very excited and interested to hear valuable opinions from you regarding the total community-based sanitation program (STBM) and access to sanitation and clean water.

- The purpose of this study was to obtain information on strategies to understand how to practice sanitation, in this case how the participation of friends with disabilities in sanitation services, especially in the Provinces of NTB and NTT. Then how the access so far that friends feel. Apart from that, we also want to know what obstacles and opportunities we can take advantage of so that the participation of this disability group can be actively involved in STBM, so that from your information, we can formulate recommendations to improve the integration of GESI (including women and women and persons with disabilities). ) in STBM.
- The information you provide is strictly confidential with your name and will not be associated with anything you convey in this discussion.
- We will record this discussion, so that we can properly capture the ideas, opinions, and thoughts that we hear from this group. The recording will be deleted as soon as the transcription process is complete.
- You have the right to refuse to answer any questions and to opt out of participation in this study.
- We realize how important and confidential the information you provide is, we remind each participant to respect each other and maintain the confidentiality of the information submitted.
- If you have questions now or later after the discussion, I can be contacted immediately or please contact other research members whose names and telephone numbers are listed on the research information form.
- Please sign consent to participate in the study.

Introduction

1. Welcome!

Introduce yourself and the minutes, quickly hand out the consent signature sheets and the demographics of the FGD participants while you introduce the discussion.

*Review*:

- Who we are and what we will do
- What we will do with the information collected
- Why we ask you to participate

1. Explanation of the discussion process

Ask if anyone has participated in a focus discussion before, explain why discussion is used instead of in-depth interview

*About discussion:*

- We learn from you (positive and negative)
- Don't try to reach a consensus, we collect information
- Don't be afraid to say a lot or be lengthy or give a long list, we will prioritize the information collected

*Logistics:*

- The discussion will last approximately one hour
- Please change places , sit and stand
- The exit! Restroom!
- Please enjoy the snacks provided

1. Basic rules of discussion

Ask participants to suggest rules of the game, give ample opportunity but make sure the following is on the list:

- Everyone must participate
- Information provided by each participant is confidential
- Stay active in the discussion but do not talk separately from one another
- Turn off the cellphone if possible
- Have fun

1. Turn on the recorder
2. Ask if there are any questions before starting
3. The discussion begins, make sure participants have enough time to think about the answers to the questions asked, don't switch participants or questions too quickly. Use probing to obtain a complete and adequate response, please change topics if the same answer is given repeatedly.

Questions for the Disability Group

ACCESS USE OF SANITATION FACILITIES AT HOME

We would like to know about the toilet and hand washing facilities in your home, their use and maintenance.

1. Do you have a toilet and handwashing station in your house that you can use easily? Can you explain? (show the demonstration picture)
   1. Who has easy access to use the toilet and hand washing station?
   2. Is there anyone in your family who can't use the toilet easily? (Who and why?)
2. Did you build the latrine/toilet yourself? Can you explain?
   1. Did you receive financial or other assistance to build your sanitation facility, or did you help build sanitation facilities for your family?
   2. How do you buy materials and construct toilet buildings? What do you think can make it easier to build a toilet?
   3. What was holding you back at that time? Why does that happen?
   4. Are you involved in deciding on the shape and how to access the existing toilets and hand washing stations?
3. Is the toilet suitable for your needs? Why?
   1. How was it before? Does anything need to be adjusted?
   2. If you look at your special needs, what things need to be different from 'regular' sanitation facilities?
   3. Who was involved in the discussion about your needs at that time?
4. Does your toilet have a hand washing area? Has it met your needs?
   1. Has it been easy to wash your hands with soap after defecating at home? Why?
   2. Do you still always wash your hands with soap after using the toilet? If not, why?
   3. What about the availability of soap at home (who has been keeping soap available all this time?)
5. Are the toilets and hand washing facilities still in use?
   1. Why is it still used/why not used? (probing: latrine quality, comfort, ease of use, availability of water, etc.)
6. In your opinion, who in your family has difficulty using toilet facilities and washing hands?
   1. Probing: (Women in the late stages of their pregnancy, elderly, People with physical disabilities, Children)
   2. How do you feel about this statement? Persons with disabilities find it more difficult to access sanitation services (toilet, hand washing facilities).
   3. For women with disabilities, what about menstrual hygiene? Are there any difficulties in doing it at home? What can make it easier/lighten?
7. What do you think is the benefit of everyone in your household using the toilet?
   1. Probing: Improving the health, hygiene and quality of life of all people, Maintaining a sense of dignity, security and privacy of people, Improving the health and hygiene of people who have to care for children, the elderly and people with disabilities in the household, Improving independence of the elderly and persons with disabilities if they are able to use their own toilet and therefore reduce daily time and physical burden on caregivers, Give primary caregivers more time to engage in other productive activities for the family
8. What is your view on toilet hygiene? Is it important?
   1. How can you explain?
   2. Who has been in charge of cleaning the toilet in your house? Why?
9. Have you ever repaired a toilet? Why?
   1. If so, what kind of repair or improvement?
   2. How did you do it?

SANITATION PROGRAM IN THE VILLAGE AND THE INVOLVEMENT OF PARTIES

We also want to know about the sanitation programs that have been carried out in your village and who was involved

(ethno biographies: tracing the timeline and program activities plenary paper tools)

1. Have you ever heard of the STBM program or other sanitation programs?
   1. Like what and who implements the sanitation program?
2. What are the targets of the program? Do you think there are other aspects that also need to be included in the sanitation program? Why?
   1. Probing: Toilet facilities, hand washing facilities, water availability, access for all
3. How important is the existence of this sanitation program to you? Why? How important is the program to your village?
4. Can you mention what activities in the sanitation program were carried out at that time?
   1. Who from the community is involved in each of these activities? Pocket voting the level of involvement of various parties in the sanitation program
   2. Have you ever been invited, involved, invited to discuss in the program? Could you please tell us what and how did you get involved in the program? Why?
   3. Are you satisfied with the engagement? Why?
   4. In your opinion, what has been done in this activity to reduce inequality for persons with disabilities? Does it work enough in your opinion? Why?
   5. In your view, have the sanitation program activities been inclusive and responsive to the needs of people with disabilities?
   6. When and why do you think gender and disability issues need to be highlighted and the stages of the sanitation program? Will it make a difference for you?
   7. In your opinion, who in your village has very little involvement? Who does not benefit from the existing sanitation program? Why?
5. What do you think is preventing you or other disabled colleagues from being involved in sanitation programs?
6. Do you think you have the same amount of power in the decision-making process as anyone else in the program? pocket voting power in decision making
7. If you were involved in a sanitation program, could your involvement change the treatment of persons with disabilities to have a toilet and use a toilet easily? (Can people with disabilities lead easy changes for groups with disabilities in sanitation programs?)
8. In your opinion, which activities in the sanitation program did you enjoy and remember the most? Why? Which activity do you dislike? Why?
9. In your opinion, how to increase the involvement and benefits of persons with disabilities in sanitation programs?

CHANGES AND CURRENT SANITATION CONDITIONS IN THE COMMUNITY

We would also like to know about the changes caused by the sanitation program in your village.

1. Can you tell us how the sanitation situation was before the sanitation program was implemented in your village?
   1. How has toilet use developed since the sanitation program?
   2. What is your current opinion about open defecation?
   3. How did you feel when you were pushed to build a toilet?
2. How did you feel when your village was declared ODF? Is that important to you?
   1. Do you think that currently in your village there are really no people who open defecation? How many people in your village do you believe use the latrine? Pocket Vote! Why?
   2. What about the kids? people with disabilities? elderly? Home location?
   3. Do you think that everyone in your village washes their hands with soap after defecating? Pocket Vote! Why?
3. Were there any other sanitation related activities carried out in your village after your village was declared Open Defecation Free (ODF)? What activities, by whom and how?
   1. Is the government doing something about it?
4. One of the reasons for the government to implement STBM is to protect children from the risk of getting sick to death, especially from diarrhea. After this program was implemented, did anyone feel any changes, especially in the frequency of sick/dead children?

**Stakeholders Interview Guidelines**

Variables or factors that inhibit or support the level of participation will be extracted from the interviews and FGDs

health/community cadre, community formal and informal leaders, community facilitators (local NGOs), government staff and decision makers (at least ten informants)

Informants Demographics

Name :

Occupation/ Position :

How long have you been in that position?

Sanitation Program that has been running in the community

1. Can you tell us about what sanitation programs have been implemented in this village/region?
   1. What is it like and who implements the program?
2. WhatWhat is the target of the program? Do you think there are other aspects that should also be included in the sanitation program? Why?
   1. Probing: Toilet facilities, hand washing facilities, water availability,
   2. What about access for all, especially for marginal groups?
   3. What about menstrual hygiene?
3. How important is the existence of this sanitation program for this village/region? what?
4. What activities are carried out in the sanitation program? How are the parties involved in the program?
   1. Who from the community is involved in each stage of the activity? Which group has more involvement than other groups in society? Why?
   2. What is the form of involvement of each community group?
   3. Are you satisfied with the level of involvement of all these community groups? Why? Which groups do you think still need to increase the level of participation?
   4. In your opinion, what has been done in these activities to reduce inequality for persons with disabilities, for women and other marginalized groups? Does it work enough in your opinion? Why?
   5. In your view, have the sanitation program activities been inclusive and responsive to the needs of people with disabilities? What about the needs of women's groups?
   6. When and why do you think gender and disability issues need to be highlighted and the stages of the sanitation program? Will it make a difference to your village/area?
   7. In your opinion, who in your village/region does not benefit from the sanitation program that is already running? Why?
   8. In your opinion, which activities in the sanitation program can best embrace the involvement of marginalized groups? Why?
   9. Which activities in the sanitation program were less successful in involving marginal groups in society in your opinion? Why?

Involvement of disability groups

1. What do you think is preventing people with disabilities from participating in sanitation programs?
2. In your opinion, do people with disabilities have the same amount of power in the decision-making process in sanitation programs? Why?
   1. Can the involvement of disability groups in sanitation programs improve access to sanitation facilities by disabled groups in your village/area?
   2. Can people with disabilities lead changes that make it easier for people with disabilities in sanitation programs?)
3. In your opinion, how do you increase the involvement and benefits of people with disabilities in sanitation programs?

**Involvement of Women**

1. What do you think is preventing women's groups from participating in sanitation programs?
2. In your opinion, do women's groups have the same amount of power in the decision-making process in sanitation programmes? Why?
   1. Can women speak and be heard in community sanitation meetings? Why? How is the process?
   2. Do you think that women can be leaders in making decisions regarding STBM in the community?
   3. Is it currently felt that women already have a role/can make decisions that are taken into account in STBM activities in the community? If so, is there anything that needs to be improved? If not, what needs to be changed so that the role of women can be increased?
   4. What factors hinder women's participation in decision-making regarding the provision of women-friendly sanitation facilities in the community?
3. In your opinion, how should the participation of women and men in the community in the provision of sanitation facilities be? Do you think the role being played is fair? Or is there something that needs to be improved? How?

Changes and sustainability of sanitation in the community

1. Can you tell us how the sanitation was before the sanitation program was implemented in your village/area?
   1. How has toilet use developed since the sanitation program?
   2. What do you think about the current public opinion about open defecation?
2. What are the achievements of ODF in this village/region? Is it important for this village/region?
   1. Do you think that currently in this village/region there are really no people who open defecation? Why?
   2. What about the kids? people with disabilities? elderly? Home location?
   3. Do you think that everyone in this village/region always washes their hands with soap after defecating? Why? Who is always and who is not?
3. One of the reasons for the government to implement STBM is to protect children from the risk of getting sick to death, especially from diarrhea. After this program has been implemented, is there a decrease in morbidity, especially related to diarrhea in children?
4. Were there any other sanitation related activities carried out in this village/area after your village was declared Open Defecation Free (ODF)? What activities, by whom and how? What is the purpose? Is there any anticipation so that people don't come back with OD?
5. What about septic tank maintenance? or toilet repair? like what has been done so far?

Policies and other supporting factors

1. What policies does your village/region have to ensure that the sanitation program implemented can benefit everyone including marginalized groups in a sustainable manner?
   1. Is there a government regulation that serves as the legal basis?
   2. Are these policies and regulations related to the SDGs?
2. Has this policy been implemented? If so, can you provide an explanation and example of its application?
   1. Who is in charge? How effective is the example?
3. Is there monitoring and evaluation of the implementation of the policy? How is the process and results monitored?
4. What are the strategies implemented in this village/region to ensure the involvement of marginalized groups in the sanitation program?
5. Do field officers or government staff have sufficient capacity to identify and facilitate the needs of marginalized groups? Like what? Why?
6. What is the role of outside organizations in implementing sanitation programs in this village/region?
   1. Has there been a change in perception regarding inclusive sanitation access in this village/region since the program?
7. What other supports are being sought to ensure sustainable access to sanitation for marginal groups in this village/region?
8. Are there risks to access to sanitation in an emergency or disaster situation?
   1. What is the impact on access to sanitation?
   2. What about access for marginalized groups?
   3. How does this village/region anticipate it?

Closing

1. Do you have any questions to ask?
2. Is there any other information you would like to disclose?
